# Genomic insights into the genetic basis of eagle‐beak jaw, large head, and long tail in the big‐headed turtle

**DOI:** 10.1002/ece3.10361

**Published:** 2023-07-25

**Authors:** Shiping Gong, Yan Ge, Yufeng Wei, Yangchun Gao

**Affiliations:** ^1^ College of Life Science and Technology Jinan University Guangzhou China; ^2^ Guangdong Key Laboratory of Animal Conservation and Resource Utilization, Guangdong Public Laboratory of Wild Animal Conservation and Utilization, Institute of Zoology Guangdong Academy of Sciences Guangzhou China

**Keywords:** eagle‐beak jaw, genetic basis, large head, long tail, *Platysternon megacephalum*

## Abstract

The big‐headed turtle (*Platysternon megacephalum*) is an endemic chelonian species in Asia. Unlike most other turtles in the world, *P. megacephalum* is characterized with eagle‐beak jaw, large head, and long tail. Although these unique characteristics are well recognized, the underlying genetic basis remains largely elusive. Here, we performed comparative genomic analysis between *P. megacephalum* and other representative species, aiming to reveal the genetic basis of the unique morphological features. Our results revealed that the eagle‐beak jaw is most likely enabled by combined effects of expansion of SFRP5, extraction of FGF11, and mutation of both ZFYVE16 and PAX6. Large head is supported by mutations of SETD2 and FGRF2 and copy number variations of six head circumference modulation‐related genes (TGFBR2, Twist2, Rdh10, Gas1, Chst11, and SNAP25). The long tail is probably involved in a genetic network comprising Gdf11, Lin 28, and HoxC12, two of which showed a consistent expression pattern with a model organism (mice). These findings suggest that expansion, extraction, and mutation of those genes may have profound effects on unique phenotypes of *P. megacephalum*.

## Graphical abstract

The genetic basis of unique phenotype characteristics (eagle‐beak jaw, large head, and long tail) in the big‐headed turtle (*Platysternon megacephalum*) is unknown. Taking comparative genome analysis and transcriptome sequencing, this study revealed several key genes with crucial roles in the development of those unique characteristics, providing insights into the genetic basis of unique phenotypes in the big‐headed turtle.

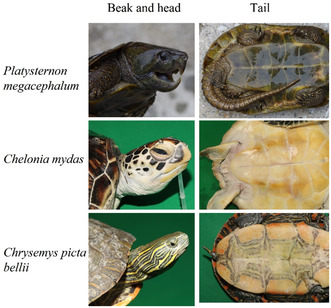

## 1 INTRODUCTION

**FIGURE 1:**
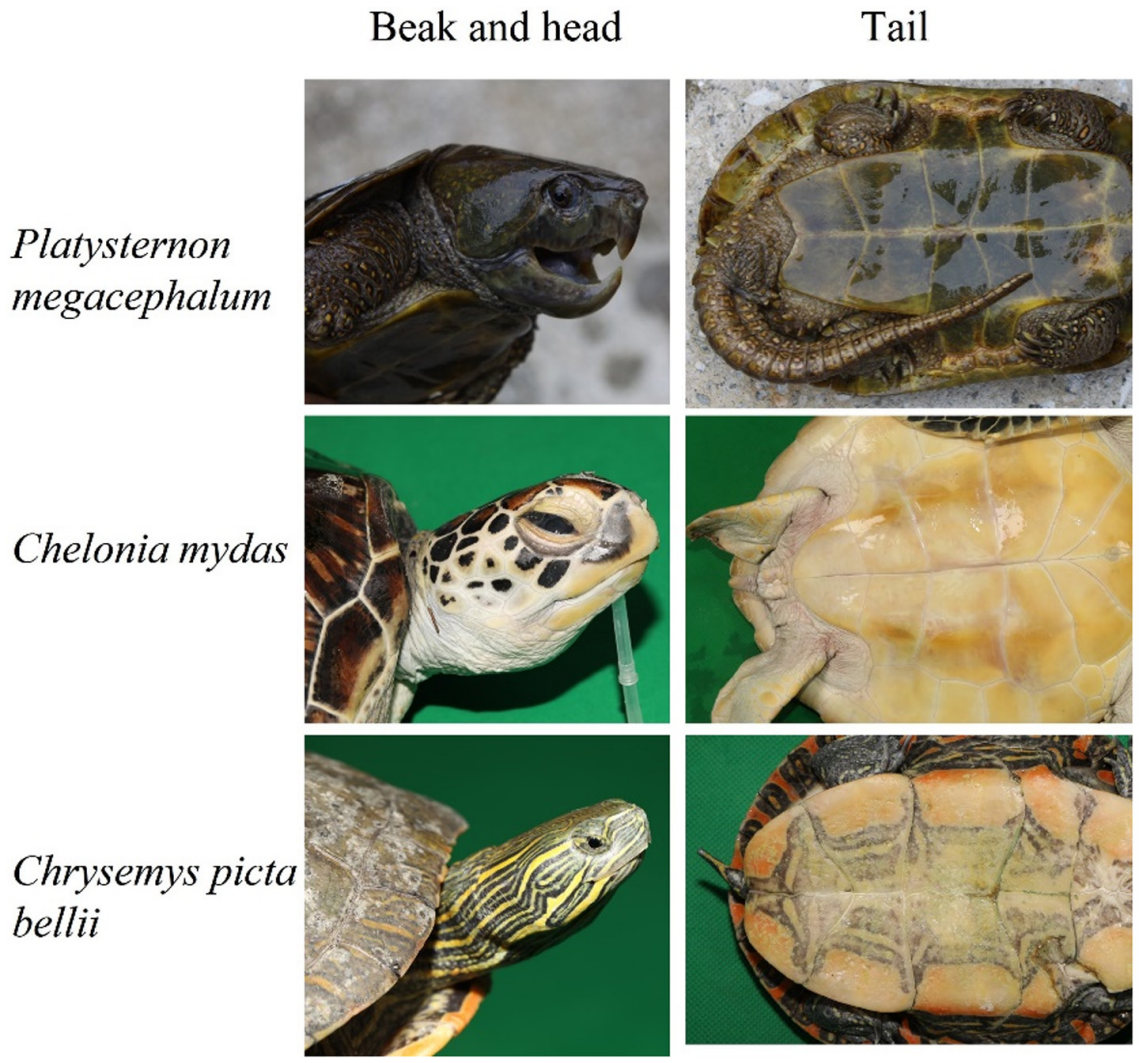
Morphological characteristics of beak, head, and tail in *Platysternon megacephalum*, *Chelonia mydas*, and *Chrysemys picta bellii*.

Most of chelonian species, such as western painted turtle (*Chrysemys picta bellii*), Chinese three‐keeled pond turtle (*Mauremys reevesii*), and red‐eared slider (*Trachemys scripta elegans*), are characterized with flat jaw, small head, and short tail compared to their body, often shrink the head and tail into the shell to avoid threats, those common freshwater turtles mainly have herbivorous or omnivorous diets in general. However, the big‐headed turtle (*Platysternon megacephalum*) in Platysternidae family, which possess carnivorous diets and inhabits mountain rocky streams, have opposite characteristics with sharp eagle‐beak hooked jaw, large head, and long tail (Figure 1). The unique characteristics in this family are regarded as adaptive phenotypes involving their carnivorous diets and strong climbing ability (Cao et al., 2019). Similar to eagle' hooked beak, such as *Falco peregrinus* and *F. cherrug*, the beak of *P. megacephalum* can also enhance their ability to hunt through effectively biting and tearing muscle or other tissues of prey, such as frogs and crabs. Large head makes it impossible to shrink into its hard shell but can contribute to their strong bite force (Cao et al., 2019). For example, crab in mountain rocky stream is one of *P. megacephalum*' major preys, *P. megacephalum* can easily destroy the crab's hard shell, that is believed to attribute to their strong bite force. Long tail is beneficial to keep body balance in hunting or climbing on steep stone. Although the unique morphological characteristics in the big‐head turtle are well recognized, the underlying genetic basis is still unknown.

Comparative genomic analysis is widely used to identify genes and biochemical pathways that underlie complex traits and adaptive processes. Multiple studies have adopted this strategy to reveal genetic variations and genes that might be crucial regulators in controlling the development of beak in birds (Zhan et al., 2013), head size in human (Bult et al., 2018; Genin et al., 2012; Haworth et al., 2019; Taal et al., 2012), and tail length in mice (Aires et al., 2019; Robinton et al., 2019). For example, sharp beak with hooked jaw is one of the most crucial morphological adaptations that allow eagles (*F. peregrinus* and *F. cherrug*) with a predatory lifestyle and carnivorous diets, key genes such as TGFB2, SCML4, and Fgf10 were identified with important roles in beak development through the comparative genomic analyses (e.g., expansion and extraction analysis) between eagles and other vertebrate species (Zhan et al., 2013). Hence, similar studies between *P. megacephalum* and other representative species from the sub‐phylum vertebrate could unveil the genetic basis of their unique morphological characteristics.

Previous studies showed that genes in three KEGG pathways (WNT, TGF‐β, and FGF) and 98 other genes play key roles in the development of avian beaks (Zhan et al., 2013), highlighting potential roles of those genes in the development of eagle‐beak jaw in *P. megacephalum*. Similarly, a total of 56 head circumference modulation‐related genes were identified in human (Bult et al., 2018; Genin et al., 2012; Haworth et al., 2019; Taal et al., 2012), suggesting that these head circumference modulation‐related genes may be candidate regulators for the large head in *P. megacephalum*. Moreover, available evidence highlighted the importance of a genetic network comprising let‐7, Gdf11, Lin 28, and Hox13 genes in controlling tail length of mice (Aires et al., 2019; Robinton et al., 2019). The observation suggested that these tail development‐related genes may be also important in regulating tail length in other vertebrate species, such as *P. megacephalum* in the present study. By comparing above phenotype variation‐correlated genes, we would get insights into the reliable genetic basis of eagle‐beak jaw, large head, and long tail in *P. megacephalum*.

Although a high‐quality genome of *P. megacephalum* with a total length of 2.32 Gb was assembled and characterized in our previous study (Cao et al., 2019), the genetic basis of unique morphological characteristics has not been explored. In addition, the genomes of *C. picta bellii*, *Chelonia mydas*, *F. peregrinus*, and *F. cherrug* have been well assembled and annotated (Shaffer et al., 2013; Wang et al., 2013; Zhan et al., 2013), thus providing an opportunity to pinpoint the genetic basis of the unique morphological characteristics in *P. megacephalum*. In this study, we performed comparative genomic analysis between *P. megacephalum* and other 14 representative vertebrate species, especially for the four phenotype similar or opposite species (*C. picta bellii*, *C. mydas*, *F. peregrinus*, and *F. cherrug*), mainly focusing on available well‐studied phenotype variation‐correlated genes, to reveal the molecular mechanism underlying eagle‐beak jaw, large head, and long tail, facilitating our understanding of the evolution of unique phenotypes in *P. megacephalum*.

## 2 MATERIALS AND METHODS

### 2.1 Gene family clustering analysis

To unravel the genetic basis of unique morphological characteristics in *P. megacephalum*, we firstly performed gene family clustering analysis with other 14 vertebrate species from the sub‐phylum vertebrate: *C. mydas*, *C. picta bellii*, *F. cherrug*, *F. peregrinus*, *Pelodiscus sinensis*, *Anolis carolinensis*, *Alligator mississippiensis*, *Alligator sinensis*, *Gallus gallus*, *Gekko japonicas*, *Homo sapiens*, *Ophiophagus hannah*, *Python bivittatus*, *Xenopus tropicalis*. For genes with alternative splicing variants, only the longest transcripts (≥30 amino acids) were kept as the representative. An all‐against‐all BLASTp (Kent, 2002) was employed to identify the similarities among the filtered sequences in *P. megacephalum* and other 14 vertebrate species with an E‐value cut‐off of 1e‐7. Gene family clustering analysis was performed to define orthologous genes among the 15 species based on TreeFam database (Li et al., 2006).

### 2.2 Phylogeny and divergence time estimation

We performed a phylogenetic analysis to estimate the phylogenetic relationships and divergence time for the Platysternidae lineage. Protein sequences of all single‐copy gene families were retrieved from the gene family clustering analysis and used for the construction of a phylogenetic tree based on the 15 representative animal species. The protein sequences for each gene family were aligned using MUSCLE (Edgar, 2004) and gaps were trimmed using Gblocks (Talavera & Castresana, 2007). Then, a super alignment matrix was achieved through concatenating the alignments. The best fit model for amino acid replacement was selected using jModelTest software (Posada, 2008) and a maximum likelihood tree was constructed using RAxML software (Stamatakis et al., 2004). The robustness of the maximum likelihood tree was assessed using the bootstrap method with 1000 replicates. Divergence time between species was estimated using mcmctree in PAML (Yang, 2007) with the parameters of “JC69 model, clock = 31,000 burn‐in and 20,000 generations' MCMC,” and nine calibration points from TimeTree (Hedges et al., 2006) were used to calibrate the divergence time.

### 2.3 Gene family expansion and contraction analyses

The evolutionary dynamics of orthologous gene families were analyzed to identify gene families that have expanded or contracted using CAFÉ v2.2 (De Bie et al., 2006) under a stochastic birth and death model. CAFÉ software could estimate global parameter λ, which represented the birth and death rate of all gene families, based on the phylogenetic tree and the datasets of gene family clustering. The significantly expanded or contracted gene families were identified with an exact *p*‐value threshold of .05, calculated by the Viterbi method in CAFÉ. Based on the significantly expanded or contracted gene families, enrichment analyses of Kyoto Encyclopedia of Genes and Genomes (KEGG) pathways were performed with FDR < 0.05 to understand high‐level functions and utilities of these significantly changed gene families.

### 2.4 Beak and large head analyses

Beak is composed of skeletal and connective tissues, outer cornified layers, olfactory epithelium, blood vessels and voluntary muscles, and oral cavity; those complex components are structured by neural crest cells, facial ectoderm, nasal placodes, paraxial mesoderm, and pharyngeal endoderm, respectively (Schneider & Helms, 2003). Beak morphology could be influenced by any of those components through local molecular signaling pathways and related genes, analysis focusing on these genes would provide insights into beak morphological innovation. For beak analysis, genes in three beak development‐related pathways (WNT, TGF‐β, and FGF) and 98 other beak development‐related genes (Zhan et al., 2013) were analyzed in five representative species selected from the above phylogenetic tree: *P. megacephalum*, *C. picta bellii*, *C. mydas*, and two eagle species (*F. peregrinus* and *F. cherrug*). Similarly, for head analysis, a total of 56 head size‐related genes (Table S1) were analyzed in these representative species (Bult et al., 2018; Genin et al., 2012; Haworth et al., 2019; Taal et al., 2012).

Briefly, reference protein sequences of those target genes were retrieved from GenBank, and subsequently aligned to the protein sets of each species using MUSCLE (Edgar, 2004) with the default parameters. We only retained the best hits determined by the program in each species for further analysis. Moreover, from the best hits, we also removed genes without a complete ORF and those with less than two neighboring genes in synteny blocks among these species. Based on the alignments, we could identify SNPs and other variable loci such as indels between those orthologous genes across species. The effects of these variable loci on protein function were assessed using protein variation effect analyzer (PROVEAN) based on sequence homology (de Brevern et al., 2012) and sorts intolerant from tolerant (SIFT) based on sequence homology and the physical properties of amino acids (Kumar et al., 2009). To further identify expanded and contracted genes from well‐studied phenotype variation‐correlated genes in *P. megacephalum*, an all‐against‐all blastp (Kent, 2002) was employed to identify gene copy number variation with the following thresholds: E‐value ≤ E‐5, identity ≥ 40%, and coverage ≥ 80%.

### 2.5 Tail length analysis

A total of three artificial breeding *P. megacephalum* and three artificial breeding *C. picta bellii* individuals were collected from a turtle farm in Guangdong Province, China. Tail muscle was dissected from each individual and all tissue samples were frozen in liquid nitrogen and stored at −80°C until the extraction of total RNA. All these procedures were conducted in accordance with the approval of Animal Care and Welfare Committee in Institute of Zoology, Guangdong Academy of Science, China (Authorization Number: GIABR20200501, Date of approval: May 1, 2020). Total RNA was extracted from each sample according to the protocol described (Gao et al., 2021). RNA sequencing libraries were constructed using the NEBNext mRNA library Prep Master Mix Set for Illumina according to the manufacturer's instructions. The libraries were sequenced with a pair end of 150 bp strategy using the Illumina NovaSeq 6000 platform.

Raw reads were firstly quality‐filtered with fastp (Chen et al., 2018) using default settings to remove adaptors, contaminants, and low‐quality reads. The obtained clean reads of *P. megacephalum* samples and *C. picta bellii* samples were then mapped to the big‐headed turtle reference genome (Cao et al., 2019) and *C. picta bellii* reference genome (Shaffer et al., 2013) using Hisat2 (Kim et al., 2019), respectively. The expression level of each gene was normalized with the value of reads per kilobase of transcript per million mapped reads (RPKM). Differently expressed genes (DEGs) were identified by comparing RPKM values between *P. megacephalum* and *C. picta bellii* using the DESeq2 program package (Anders & Huber, 2010), and genes with fold changes >2 and an adjusted *p‐*value (*p*adj) < .05 were assigned as DEGs. From the DEGs, we mainly focusing on the expression levels of tail development‐related genes (Gdf11, Lin 28, Hox13, and let‐7), so as to get reliable results.

## 3 RESULTS

### 3.1 Genome comparison

**FIGURE 2:**
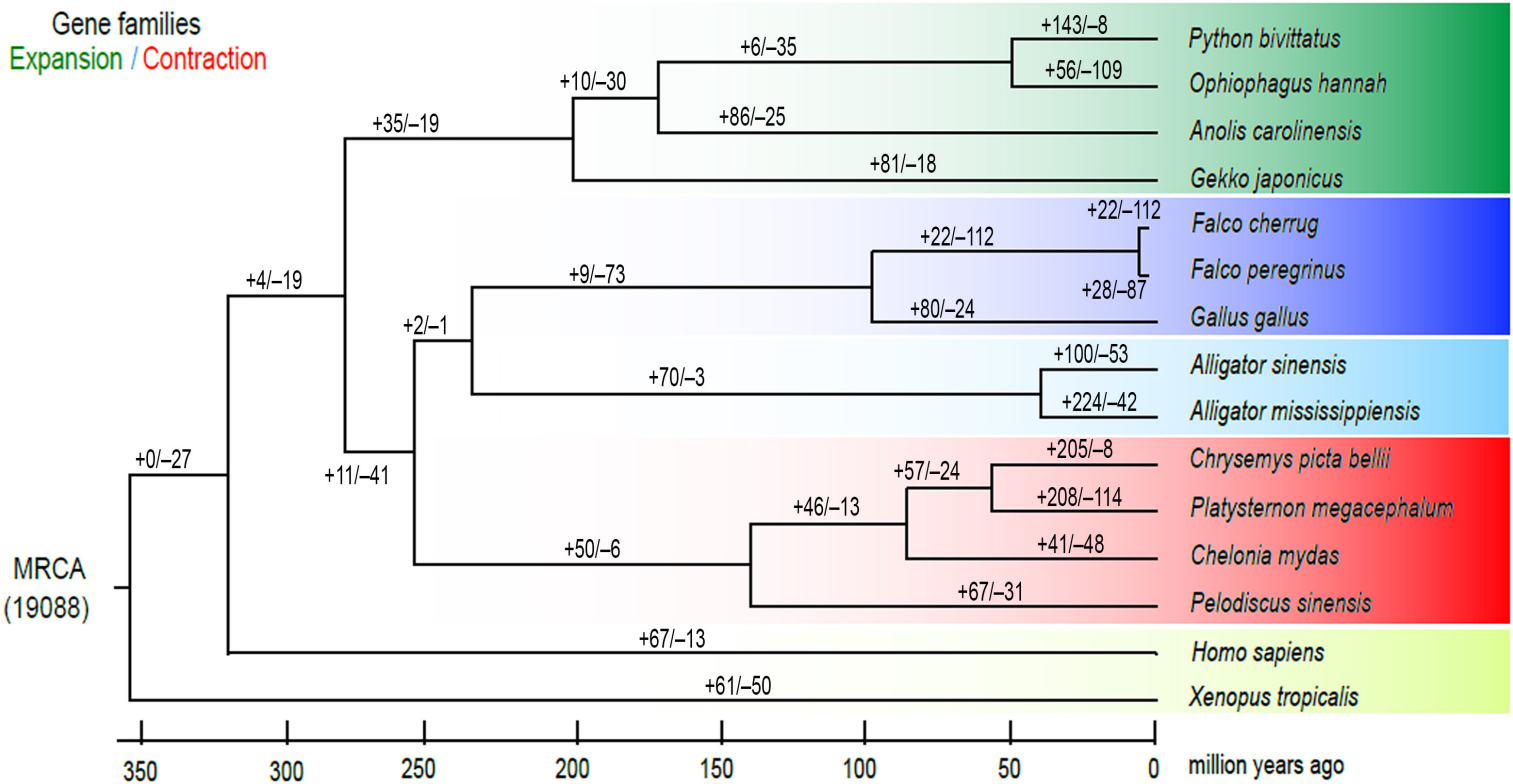
Gene family expansion/contraction analysis based on the most recent common ancestor (MRCA) of 19,088 gene families for 15 vertebrate animal species.

Gene family clustering analysis reveals 5971 shared gene families, among which 3276 are highly conserved, single‐copy orthologous genes. Phylogenetic analysis based on the highly conserved orthologous genes suggests that *P. megacephalum* diverged around ~55 Ma from the lineage leading to *C. picta* bellii (Figure S1). In addition, based on the sister taxon relationship between freshwater turtle and marine turtle, our phylogenetic analysis gives an estimation of 83 Ma for the appearance of the freshwater turtle lineage or its divergence from the marine turtle lineage (Figure S1). Compared with the most recent common ancestor (19,088 gene families), 208 expanded gene families are identified in the big‐headed turtle lineage (Figure 2) with notable enrichment of RIG‐I‐like receptor signaling pathway (Table S2).

### 3.2 The genetic basis of eagle‐beak jaw

Beak analysis revealed eight significantly expanded genes (SFRP5, LRP5, MAPK3, FGF13, FGF14, FGFR3, NAB1, and RGCC) and one significantly contracted gene (FGF11) in *P. megacephalum* genome, compared to other common turtles (*C. picta bellii* and *C. mydas*, Table 1). Among these genes in *P. megacephalum* genome, SFRP5 with two copies (SFRP5 and SFRP5‐like) was also detected in genomes of two eagles *F. peregrinus* and *F. cherrug* (Table 1). The only contracted gene FGF11 was not detected in *P. megacephalum*, *F. peregrinus*, and *F. cherrug*, but detected with a single copy in *C. picta bellii* and *C. mydas* (Table 1). In addition, sequence alignment analysis revealed 56 beak development‐related genes with the same variable loci among sharp beak species (*P. megacephalum*, *F. peregrinus*, and *F. cherrug*), but different from flat beak species (*C. picta* and *C. mydas*; Table 1). Specifically, the mutation of ZFYVE16, indels of 14 amino acids in PAX6 and 177 amino acids in SMAD7 could influence the function of these genes, respectively.

**TABLE 1:** Expanded and contracted genes involved in the development of beak in three carnivorous species (*Platysternon megacephalum*, *Falco peregrinus*, and *Falco cherrug*) and two omnivorous species (*Chrysemys picta bellii* and *Chelonia mydas*).

Gene	Pathways	*Platysternon megacephalum*	*Chrysemys picta bellii*	*Chelonia mydas*	*Falco peregrinus*	*Falco cherrug*
SFRP5	WNT	evm.model.scaf90.2 evm.model.scaf30.85	XP_008171549.1	XP_007069596.1	XM_005238947.1 XM_005230048.1	XM_014276756.1 XM_005432627.1
LRP5		evm.model.scaf287.3 evm.model.scaf287.5	XP_008171240.1	XP_007056798.1	XM_013298249.1	XM_014282838.1
MAPK3	TGFB	evm.model.scaf1445.2 evm.model.scaf753.1	XP_005290239.1	XP_007072792.1	None	None
FGF11	FGF	None	XP_005314407.1	XP_007053059.1	None	None
FGF13		evm.model.scaf5.102 evm.model.scaf5.103	XP_008177231.1	XP_007060142.1	XM_005232749.2	XM_005436083.1
FGF14		evm.model.scaf10.16 evm.model.scaf10.13	XP_005303453.1	XP_007066300.1	XM_005229397.1	XM_005440929.1
FGFR3		evm.model.scaf46.60.1 evm.model.scaf903.1	XP_005292454.1	XP_007056030.1	XM_005229861.2	XM_005446432.2
NAB1	Others	evm.model.scaf15.22 evm.model.scaf15.23 evm.model.scaf17325.1	XP_005308064.1	XP_007069947.1	XM_005241311.1	XM_005440460.1
RGCC		evm.model.scaf2.279 evm.model.scaf26779.1 evm.model.scaf294.10	XP_005301539.1 XP_005279215.1	XP_007061389.1 XP_007064037.1	XM_013297448.1	XM_014282187.1

### 3.3 The genetic basis of large head

**FIGURE 3:**
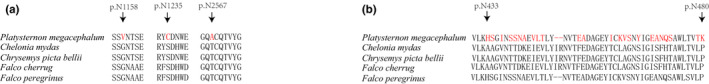
Alignment of SETD2 residue p.N1158, p.N1235, p.N2567 (a) and FGFR2 residue p.N433‐480 (b) in *Platysternon megacephalum*, *Falco cherrug*, *Falco peregrinus*, *Chelonia mydas*, *Chrysemys picta bellii*.

Head analysis revealed two genes, SETD2 and FGFR2, with specific mutations in *P. megacephalum* relative to other species (Figure 2a). We found that SETD2 in *P. megacephalum* has three mutations at position 3473 (G–T), 3704 (C–G), and 7699 (A–G), compared to other turtles and eagle species. The three mutations lead to changes in amino acids, mainly from Glycine (G) to Valine (V) at position p.N1158, Serine (S) to Cysteine (C) at p.N1235, and Threonine (T) to Alanine (A) at p.N2567, respectively. Interestingly, those amino acid changes were all *P. megacephalum*‐specific among turtle species (Figure 3a). Although FGFR2 displayed no species‐specific mutations in *P. megacephalum* genome compared to other turtles globally, one region ranging from position 1294 to 1440 showed a high mutation rate compared to other turtle species (*C. mydas* and *C. picta bellii*) with relatively small head, and the region consists of 48 amino acids, among which 26 amino acids (54.17%) were changed or deleted (Figure 3b).

**FIGURE 4:**
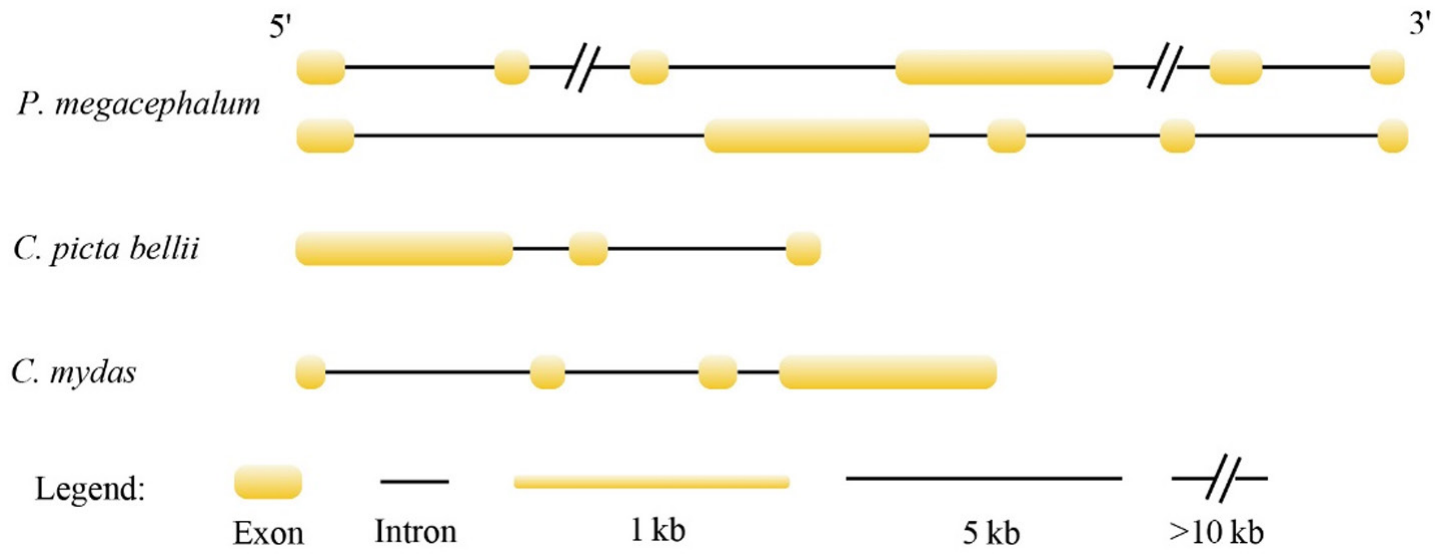
TGFBR2 gene structures of *Platysternon megacephalum*, *Falco cherrug*, *Falco peregrinus*, *Chelonia mydas*, *Chrysemys picta bellii*.

Moreover, we identified six significantly expanded head circumference modulation‐related genes (TGFBR2, Twist2, Rdh10, Gas1, Chst11, SNAP25) with two copies in *P. megacephalum* genome, compared to only one gene copy in other turtles (*C. mydas* and *C. picta bellii*) with relatively small heads (Table 2). TGFBR2 in *P. megacephalum* has more exons (five or six exons) than that in *C. mydas* and *C. picta bellii* (three or four exons, Figure 4). Whereas GAS1 in *P. megacephalum* has only one exon for each copy, less than three exons in phylogenetic closely species *C. picta bellii* (Table 2). The three of rest four genes (Twist2, Rdh10, and SNAP25) also showed variations in exon number between *P. megacephalum* and the other turtles (*C. mydas* and *C. picta bellii*, Table 2). Specifically, GAS1 was detected with two introns in *C. picta bellii*, whereas both copies of GAS1 in *P. megacephalum* were all intronless.

**TABLE 2:** CDS number of head size‐related genes in *Platysternon megacephalum*, *Chelonia mydas*, and *Chrysemys picta bellii*.

Gene	*Platysternon megacephalum* (2 copy)	*Chelonia mydas* (1 copy)	*Chrysemys picta bellii* (1 copy)
CHST11	1/1	1	1
GAS1	1/1	0	3
RDH10	4/6	4	4
TGFBR2	5/6	4	3
TWIST2	1/1	1	1
SNAP25	4/3	4	4

### 3.4 The genetic basis of long tail

To reveal the genetic basis of long tail in *P. megacephalum*, we performed transcriptome sequencing of tail muscle in *P. megacephalum* and one representative turtle species *C. picta bellii* with a short tail. A total of 2928 DEGs were identified based on tail muscle samples of two species. Among four tail development‐related genes (Gdf11, Lin 28, Hox13, and let‐7), only Gdf11 was identified as DEG, which displayed 9.2‐fold downregulated expression with a significant difference (*p*adj < .01) in *P. megacephalum* compared to that in *C. picta bellii*. Moreover, although the expression of Hox13 showed no significant difference (*p*adj > .05) between the two species, its homologous gene (HoxC12) in *P. megacephalum* showed 5.6‐fold downregulated expression with a significant difference (*p*adj < .01) compared to that in *C. picta bellii*.

## 4 DISCUSSION

We identified 208 expanded gene families in the genome of *P. megacephalum*, those expanded gene families may help *P. megacephalum* to cope with complex environmental conditions more efficiently, which may be a part of *P. megacephalum* adaptation to environmental changes through long‐term evolutionary history. Specifically, the significant enrichment of RIG‐I‐like receptor signaling pathway based on 10 immunity‐associated expanded genes in *P. megacephalum* may highlight the turtle's more developed immune systems than other vertebrate species (Table S2). The more developed immune systems were also detected in tortoise species such as *Chelonoidis abingdonii* and *Aldabrachelys gigantea* (Quesada et al., 2019), suggesting the enhancement of immune systems may correlate with the extended lifespan of these turtle and tortoise species.

### 4.1 Eagle‐beak regulation

*Platysternon megacephalum* has a remarkably eagle‐beak jaw compared to most of other herbivorous or omnivorous turtles such as *C. picta bellii* and *C. mydas*, probably as adaptation to carnivorous diet (Bonin et al., 2006). The sharp beak can benefit *P. megacephalum* hunting and teasing frog and other prey's muscle tissues. Gene copy number variants are thought to affect gene expression through altering gene dosage or modifying the chromatin environment in the vicinity of gene copy number variants (Kleinjan & van Heyningen, 2005). Therefore, gene copy number variants (e.g., expanded genes and contracted genes) may potentially play crucial roles in morphological innovation. In the present study, eight significantly expanded genes (SFRP5, LRP5, MAPK3, FGF13, FGF14, FGFR3, NAB1, and RGCC) and one significantly contracted gene (FGF11) were detected in *P. megacephalum* genome, compared to *C. picta bellii* and *C. mydas*, suggesting those genes may play crucial roles in the formation of sharp beak in *P. megacephalum*.

Interestingly, SFRP5 with two copies (SFRP5 and SFRP5‐like) was also detected in genomes of two eagles *F. peregrinus* and *F. cherrug*, both are raptors and have a predatory lifestyle and carnivorous diet with similar sharp hooked beaks (Zhan et al., 2013). In addition, the only contracted gene FGF11 was not detected in species with sharp beaks, but detected with a single copy in species with flat beaks, highlighting the importance of copy number variations of SFRP5 and FGF11 in regulating the development of eagle‐beak jaw in *P. megacephalum*. Moreover, we also identified 56 beak development‐related genes with the same variable loci among sharp beak species (*P. megacephalum*, *F. peregrinus*, and *F. cherrug*), but different from flat beak species (*C. picta* and *C. mydas*). Among those genes, the genetic variation of three genes (ZFYVE16, PAX6, and SMAD7) can influence their function, also suggested the crucial roles of genetic variation of these genes in the formation of eagle‐beak jaw in *P. megacephalum*.

### 4.2 Large head evolution

*Platysternon megacephalum* possesses a large head and thus are thought to have strong bite force, probably as adaptation to carnivorous diet as well (Bonin et al., 2006). Based on 56 genes classified in a well‐established census of genes as head circumference modulation (Bult et al., 2018; Genin et al., 2012; Haworth et al., 2019; Taal et al., 2012), we identified two head circumference regulation genes, SETD2 and FGFR2, with mutations in *P. megacephalum*. SETD2 is a histone methyltransferase and non‐redundantly responsible for all trimethylation of lysine 36 of histone H3 (Edmunds et al., 2008). The mutations in SETD2 cause a novel overgrowth condition in human, leading to Sotos syndrome, which was typically characterized by macrocephaly (Luscan et al., 2014). We found that the big‐headed turtle's SETD2 has three mutations, which can lead to changes in amino acids. Interestingly, those amino acid changes were all *P. megacephalum*‐specific among turtle species. These results suggested that SETD2 may play a key role in regulating head circumference development in *P. megacephalum*. Furthermore, FGFR2 is a transmembrane receptor with a tyrosine kinase domain, a transmembrane region, and an extracellular ligand‐binding regions. Mutations in FRFR2 significantly contribute to disorders of bone growth and development, leading to Pfeiffer syndrome, which is mainly characterized by craniosynostosis and great bone‐related morphology (Júnior et al., 2015; Machado et al., 2017). Although FGFR2 displayed no species‐specific mutations in *P. megacephalum* genome compared to other turtles globally, one region ranging from position 1294 to 1440 showed a high mutation rate (54.17%) compared to *C. mydas* and *C. picta bellii* with relatively small head. The above results indicated that mutations in SETD2 and FGRF2 may be one of mechanisms underlying large head regulation in *P. megacephalum*.

Moreover, six significantly expanded head circumference modulation‐related genes (TGFBR2, Twist2, Rdh10, Gas1, Chst11, and SNAP25) were detected in *P. megacephalum* genome. Specifically, TGFBR2 has more exons (five or six exons) and GAS1 has less exon (only one exon) in *P. megacephalum* compared to other turtles. Variation in exon number was also detected in the three of rest four genes (Twist2, Rdh10, and SNAP25), suggesting that the variation in exon number of head circumference modulation‐related genes highlighted the importance of those genes in regulating the development of large head in *P. megacephalum*, as inferred from the crucial role of variation in exon number in organ development or disease occurrence (Kim et al., 2020). The occurrence of intronless genes suggested high transcription efficiency due to no need of post‐transcriptional splicing (De Renzis et al., 2007; Li et al., 2017). The utilization of intronless GAS1 in *P. megacephalum* may represent an adaptive change to enhance transcription efficiency to facilitate the large head. The six genes combined with SETD2 and FGFR2 were all involved in embryonic cranial skeleton morphogenesis, which displayed close relationship with morphogenesis of head bones (Bult et al., 2018; Opperman et al., 2005), indicating that gene copy number variants or mutation in these genes may contribute to *P. megacephalum* with large head.

### 4.3 Long tail regulation

Tail length is positively correlated with caudal vertebrae number and tail bud progenitor cell proliferation and specification in vertebrate organism (Bénazéraf & Pourquié, 2013). One recent study on mice showed that caudal vertebrae number and tail bud cell proliferation were largely controlled by the heterochronic genes Lin28a/b and let‐7, overexpression of which remarkably increased tail bud cell proliferation and caudal vertebrae number, and thus led to long tail, whereas knockout of these genes led to opposite effects (Robinton et al., 2019). A similar study revealed that inhibition of Gdf11 gene could increase number of tail bud progenitors by activating Hox13 gene, which could downregulate the expression of Lin28 genes, highlighting the importance of a genetic network comprising Gdf11, Lin 28, and Hox13 genes in influencing tail bud progenitor activity and controlling tail length (Aires et al., 2019). Long tail is one of the most important characteristics in the big‐headed turtle and plays a key role in keeping body balance when they fight against other individuals or climb on the steep rock habitats. Our study based on comparative transcriptome sequencing of tail showed that both Gdf11 and HoxC12 (Hox13 homologous gene) genes in *P. megacephalum* with a long tail displayed downregulated expressions compared to that in *C. picta bellii* with a short tail, suggesting Gdf11 and HoxC12 may be negative regulators in the development of tail in *P. megacephalum*. Hence, we propose that decreased expression of Gdf11 in *P. megacephalum* inhibits the expression of HoxC12, which further upregulates the expression of Lin 28 and thus increases number of tail bud progenitors, ultimately leading to long tail in *P. megacephalum*.

## AUTHOR CONTRIBUTIONS

**Shiping Gong:** Conceptualization (equal); data curation (equal); funding acquisition (equal); investigation (equal); resources (equal); writing – review and editing (equal). **Yan Ge:** Resources (equal). **Yufeng Wei:** Formal analysis (equal); investigation (equal); methodology (equal); writing – review and editing (equal). **Yangchun Gao:** Conceptualization (equal); data curation (equal); funding acquisition (equal); methodology (equal); validation (equal); writing – original draft (equal); writing – review and editing (equal).

## CONFLICT OF INTEREST STATEMENT

None declared.

## Supporting information


Figure S1.

Appendix S1.

## Data Availability

Sequencing reads are available at the NCBI SRA under accession numbers: SRR23003489–SRR23003494.
