# Supplementary material for: Genomic insights into the genetic basis of eagle‐beak jaw, large head, and long tail in the big‐headed turtle
Source: Ecol Evol. 2023 Jul 25;13(7):e10361. doi: 10.1002/ece3.10361 (PMC10368965; doi:10.1002/ece3.10361)
Supplement: Supplementary file 1 — Figure S1. [file ECE3-13-e10361-s001.docx]

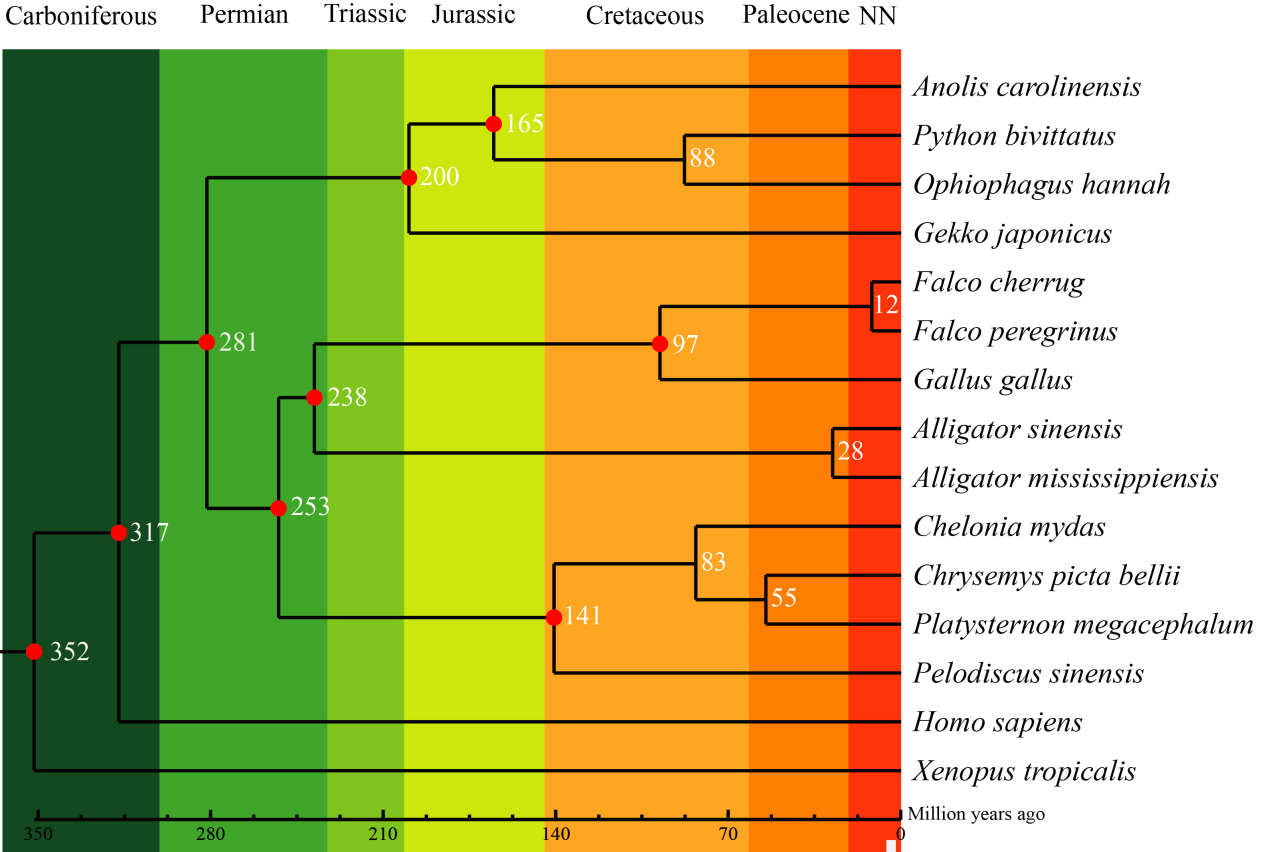


Supplementary Fig.1 Estimated divergence times of *Platysternon megacephalum* with other 14 vertebrate species using 3276 single-copy orthologous genes. The red circles indicate the fossil calibration times
