# Supplementary material for: Genomic insights into the genetic basis of eagle‐beak jaw, large head, and long tail in the big‐headed turtle
Source: Ecol Evol. 2023 Jul 25;13(7):e10361. doi: 10.1002/ece3.10361 (PMC10368965; doi:10.1002/ece3.10361)
Supplement: Supplementary file 2 — Appendix S1. [file ECE3-13-e10361-s002.docx]

Supplementary Table 1 A total of 56 head size-related genes analyzed in this study

| Entry | Gene names | Protein names | Length |
| --- | --- | --- | --- |
| O95076 | ALX3 | Homeobox protein aristaless-like 3 (Proline-rich transcription factor ALX3) | 343 |
| P05549 | TFAP2A AP2TF TFAP2 | Transcription factor AP-2-alpha (AP2-alpha) (AP-2 transcription factor) (Activating enhancer-binding protein 2-alpha) (Activator protein 2) (AP-2) | 437 |
| P12644 | BMP4 BMP2B DVR4 | Bone morphogenetic protein 4 (BMP-4) (Bone morphogenetic protein 2B) (BMP-2B) | 408 |
| Q6UB35 | MTHFD1L FTHFSDC1 | Monofunctional C1-tetrahydrofolate synthase, mitochondrial (EC 6.3.4.3) (Formyltetrahydrofolate synthetase) | 978 |
| Q9NPF2 | CHST11 | Carbohydrate sulfotransferase 11 (EC 2.8.2.5) (Chondroitin 4-O-sulfotransferase 1) (Chondroitin 4-sulfotransferase 1) (C4S-1) (C4ST-1) (C4ST1) | 352 |
| Q98875 | dlx1a dlx1 | Homeobox protein Dlx1a (DLX-1) (Distal-less homeobox gene 1a) | 252 |
| Q07687 | DLX2 | Homeobox protein DLX-2 | 328 |
| P55075 | FGF8 AIGF | Fibroblast growth factor 8 (FGF-8) (Androgen-induced growth factor) (AIGF) (Heparin-binding growth factor 8) (HBGF-8) | 233 |
| P21802 | FGFR2 BEK KGFR KSAM | Fibroblast growth factor receptor 2 (FGFR-2) (EC 2.7.10.1) (K-sam) (KGFR) (Keratinocyte growth factor receptor) (CD antigen CD332) | 821 |
| Q99958 | FOXC2 FKHL14 MFH1 | Forkhead box protein C2 (Forkhead-related protein FKHL14) (Mesenchyme fork head protein 1) (MFH-1 protein) (Transcription factor FKH-14) | 501 |
| Q9BT04 | FUZ FY | Protein fuzzy homolog | 418 |
| P54826 | GAS1 | Growth arrest-specific protein 1 (GAS-1) | 345 |
| P10071 | GLI3 | Transcriptional activator GLI3 (GLI3 form of 190 kDa) (GLI3-190) (GLI3 full-length protein) (GLI3FL) [Cleaved into: Transcriptional repressor GLI3R (GLI3 C-terminally truncated form) (GLI3 form of 83 kDa) (GLI3-83)] | 1580 |
| Q5JWF2 | GNAS GNAS1 | Guanine nucleotide-binding protein G(s) subunit alpha isoforms XLas (Adenylate cyclase-stimulating G alpha protein) (Extra large alphas protein) (XLalphas) | 1037 |
| Q6ISB3 | GRHL2 BOM TFCP2L3 | Grainyhead-like protein 2 homolog (Brother of mammalian grainyhead) (Transcription factor CP2-like 3) | 625 |
| P49639 | HOXA1 HOX1F | Homeobox protein Hox-A1 (Homeobox protein Hox-1F) | 335 |
| O43364 | HOXA2 HOX1K | Homeobox protein Hox-A2 (Homeobox protein Hox-1K) | 376 |
| Q96RY7 | IFT140 KIAA0590 WDTC2 | Intraflagellar transport protein 140 homolog (WD and tetratricopeptide repeats protein 2) | 1462 |
| P38919 | EIF4A3 DDX48 KIAA0111 | Eukaryotic initiation factor 4A-III (eIF-4A-III) (eIF4A-III) (EC 3.6.4.13) (ATP-dependent RNA helicase DDX48) (ATP-dependent RNA helicase eIF4A-3) (DEAD box protein 48) (Eukaryotic initiation factor 4A-like NUK-34) (Eukaryotic translation initiation factor 4A isoform 3) (Nuclear matrix protein 265) (NMP 265) (hNMP 265) [Cleaved into: Eukaryotic initiation factor 4A-III, N-terminally processed] | 411 |
| P48742 | LHX1 LIM-1 LIM1 | LIM/homeobox protein Lhx1 (LIM homeobox protein 1) (Homeobox protein Lim-1) (hLim-1) | 406 |
| Q93074 | MED12 ARC240 CAGH45 HOPA KIAA0192 TNRC11 TRAP230 | Mediator of RNA polymerase II transcription subunit 12 (Activator-recruited cofactor 240 kDa component) (ARC240) (CAG repeat protein 45) (Mediator complex subunit 12) (OPA-containing protein) (Thyroid hormone receptor-associated protein complex 230 kDa component) (Trap230) (Trinucleotide repeat-containing gene 11 protein) | 2177 |
| Q06413 | MEF2C | Myocyte-specific enhancer factor 2C (Myocyte enhancer factor 2C) | 473 |
| P50281 | MMP14 | Matrix metalloproteinase-14 (MMP-14) (EC 3.4.24.80) (MMP-X1) (Membrane-type matrix metalloproteinase 1) (MT-MMP 1) (MTMMP1) (Membrane-type-1 matrix metalloproteinase) (MT1-MMP) (MT1MMP) | 582 |
| P51512 | MMP16 C8orf57 MMPX2 | Matrix metalloproteinase-16 (MMP-16) (EC 3.4.24.-) (MMP-X2) (Membrane-type matrix metalloproteinase 3) (MT-MMP 3) (MTMMP3) (Membrane-type-3 matrix metalloproteinase) (MT3-MMP) (MT3MMP) | 607 |
| P52848 | NDST1 HSST HSST1 | Bifunctional heparan sulfate N-deacetylase/N-sulfotransferase 1 (EC 2.8.2.8) (Glucosaminyl N-deacetylase/N-sulfotransferase 1) (NDST-1) (N-heparan sulfate sulfotransferase 1) (N-HSST 1) ([Heparan sulfate]-glucosamine N-sulfotransferase 1) (HSNST 1) [Includes: Heparan sulfate N-deacetylase 1 (EC 3.-.-.-); Heparan sulfate N-sulfotransferase 1 (EC 2.8.2.-)] | 882 |
| Q6KC79 | NIPBL IDN3 SCC2 | Nipped-B-like protein (Delangin) (SCC2 homolog) | 2804 |
| Q96S42 | NODAL | Nodal homolog | 347 |
| Q02548 | PAX5 | Paired box protein Pax-5 (B-cell-specific transcription factor) (BSAP) | 391 |
| P16234 | PDGFRA PDGFR2 RHEPDGFRA | Platelet-derived growth factor receptor alpha (PDGF-R-alpha) (PDGFR-alpha) (EC 2.7.10.1) (Alpha platelet-derived growth factor receptor) (Alpha-type platelet-derived growth factor receptor) (CD140 antigen-like family member A) (CD140a antigen) (Platelet-derived growth factor alpha receptor) (Platelet-derived growth factor receptor 2) (PDGFR-2) (CD antigen CD140a) | 1089 |
| P54821 | PRRX1 PMX1 | Paired mesoderm homeobox protein 1 (Homeobox protein PHOX1) (Paired-related homeobox protein 1) (PRX-1) | 245 |
| Q99811 | PRRX2 PMX2 PRX2 | Paired mesoderm homeobox protein 2 (Paired-related homeobox protein 2) (PRX-2) | 253 |
| Q8IZV5 | RDH10 SDR16C4 UNQ9375/PRO34191 | Retinol dehydrogenase 10 (EC 1.1.1.300) (Short chain dehydrogenase/reductase family 16C member 4) | 341 |
| Q13950 | RUNX2 AML3 CBFA1 OSF2 PEBP2A | Runt-related transcription factor 2 (Acute myeloid leukemia 3 protein) (Core-binding factor subunit alpha-1) (CBF-alpha-1) (Oncogene AML-3) (Osteoblast-specific transcription factor 2) (OSF-2) (Polyomavirus enhancer-binding protein 2 alpha A subunit) (PEA2-alpha A) (PEBP2-alpha A) (SL3-3 enhancer factor 1 alpha A subunit) (SL3/AKV core-binding factor alpha A subunit) | 521 |
| Q9NY26 | SLC39A1 IRT1 ZIP1 ZIRTL CGI-08 CGI-71 | Zinc transporter ZIP1 (Solute carrier family 39 member 1) (Zinc-iron-regulated transporter-like) (Zrt- and Irt-like protein 1) (ZIP-1) (hZIP1) | 324 |
| Q9BRY0 | SLC39A3 ZIP3 | Zinc transporter ZIP3 (Solute carrier family 39 member 3) (Zrt- and Irt-like protein 3) (ZIP-3) | 314 |
| Q9BYW2 | SETD2 HIF1 HYPB KIAA1732 KMT3A SET2 HSPC069 | Histone-lysine N-methyltransferase SETD2 (EC 2.1.1.359) (HIF-1) (Huntingtin yeast partner B) (Huntingtin-interacting protein 1) (HIP-1) (Huntingtin-interacting protein B) (Lysine N-methyltransferase 3A) (Protein-lysine N-methyltransferase SETD2) (EC 2.1.1.-) (SET domain-containing protein 2) (hSET2) (p231HBP) | 2564 |
| Q15475 | SIX1 | Homeobox protein SIX1 (Sine oculis homeobox homolog 1) | 284 |
| Q9NPC8 | SIX2 | Homeobox protein SIX2 (Sine oculis homeobox homolog 2) | 291 |
| Q9UIU6 | SIX4 | Homeobox protein SIX4 (Sine oculis homeobox homolog 4) | 781 |
| Q15796 | SMAD2 MADH2 MADR2 | Mothers against decapentaplegic homolog 2 (MAD homolog 2) (Mothers against DPP homolog 2) (JV18-1) (Mad-related protein 2) (hMAD-2) (SMAD family member 2) (SMAD 2) (Smad2) (hSMAD2) | 467 |
| P84022 | SMAD3 MADH3 | Mothers against decapentaplegic homolog 3 (MAD homolog 3) (Mad3) (Mothers against DPP homolog 3) (hMAD-3) (JV15-2) (SMAD family member 3) (SMAD 3) (Smad3) (hSMAD3) | 425 |
| O43435 | TBX1 | T-box transcription factor TBX1 (T-box protein 1) (Testis-specific T-box protein) | 398 |
| Q96SF7 | TBX15 TBX14 | T-box transcription factor TBX15 (T-box protein 15) (T-box transcription factor TBX14) (T-box protein 14) | 602 |
| P61812 | TGFB2 | Transforming growth factor beta-2 proprotein (Cetermin) (Glioblastoma-derived T-cell suppressor factor) (G-TSF) [Cleaved into: Latency-associated peptide (LAP); Transforming growth factor beta-2 (TGF-beta-2)] | 414 |
| P10600 | TGFB3 | Transforming growth factor beta-3 proprotein [Cleaved into: Latency-associated peptide (LAP); Transforming growth factor beta-3 (TGF-beta-3)] | 412 |
| P36897 | TGFBR1 ALK5 SKR4 | TGF-beta receptor type-1 (TGFR-1) (EC 2.7.11.30) (Activin A receptor type II-like protein kinase of 53kD) (Activin receptor-like kinase 5) (ALK-5) (ALK5) (Serine/threonine-protein kinase receptor R4) (SKR4) (TGF-beta type I receptor) (Transforming growth factor-beta receptor type I) (TGF-beta receptor type I) (TbetaR-I) | 503 |
| P37173 | TGFBR2 | TGF-beta receptor type-2 (TGFR-2) (EC 2.7.11.30) (TGF-beta type II receptor) (Transforming growth factor-beta receptor type II) (TGF-beta receptor type II) (TbetaR-II) | 567 |
| O75386 | TULP3 TUBL3 | Tubby-related protein 3 (Tubby-like protein 3) | 442 |
| Q15672 | TWIST1 BHLHA38 TWIST | Twist-related protein 1 (Class A basic helix-loop-helix protein 38) (bHLHa38) (H-twist) | 202 |
| Q8WVJ9 | TWIST2 BHLHA39 DERMO1 | Twist-related protein 2 (Class A basic helix-loop-helix protein 39) (bHLHa39) (Dermis-expressed protein 1) (Dermo-1) | 160 |
| Q8NEZ3 | WDR19 IFT144 KIAA1638 | WD repeat-containing protein 19 (Intraflagellar transport 144 homolog) | 1342 |
| O14905 | WNT9B WNT14B WNT15 UNQ6973/PRO21956 | Protein Wnt-9b (Protein Wnt-14b) (Protein Wnt-15) | 357 |
| P04637 | TP53 P53 | Cellular tumor antigen p53 (Antigen NY-CO-13) (Phosphoprotein p53) (Tumor suppressor p53) | 393 |
| P51608 | MECP2 | Methyl-CpG-binding protein 2 (MeCp-2 protein) (MeCp2) | 486 |
| Q8NG31 | KNL1 CASC5 KIAA1570 | Kinetochore scaffold 1 (ALL1-fused gene from chromosome 15q14 protein) (AF15q14) (Bub-linking kinetochore protein) (Blinkin) (Cancer susceptibility candidate gene 5 protein) (Cancer/testis antigen 29) (CT29) (Kinetochore-null protein 1) (Protein CASC5) (Protein D40/AF15q14) | 2342 |
| P60880 | SNAP25 SNAP | Synaptosomal-associated protein 25 (SNAP-25) (Super protein) (SUP) (Synaptosomal-associated 25 kDa protein) | 206 |

Supplementary Table 2 KEGG enrichment analysis of gene families expanded in the *P.* *megacephalum* genome.

| MapID | MapTitle | P-value | Adjusted P-value |
| --- | --- | --- | --- |
| map04622 | RIG-I-like receptor signaling pathway | 2.74E-05 | 0.00834 |
